# Factors affecting sleep quality in hospitalised patients

**DOI:** 10.1007/s11325-024-03144-8

**Published:** 2024-09-07

**Authors:** Kavya Koshy, Matthew Gibney, Denise M. O’Driscoll, Rowan P. Ogeil, Alan C. Young

**Affiliations:** 1 Department of Respiratory and Sleep Medicine, Box Hill Hospital, Box Hill, VIC Australia; 2 Eastern Health Clinical School, Monash University, Melbourne, VIC Australia; 3 Turning Point, Eastern Health, Melbourne, VIC Australia

**Keywords:** Sleep, Circadian rhythm, Sleep disturbance, Sleep impairment

## Abstract

**Introduction:**

Hospitalised patients are at increased risk of poor sleep quality which can negatively impact on recovery and quality of life. This study aimed to assess sleep quality in hospitalised patients and explore the factors associated with poor sleep.

**Methods:**

Prospective data were collected from 84 respiratory ward inpatients at time of discharge using a Likert scale questionnaire on contributing factors to sleep quality. Differences between groups reporting good and poor quality sleep were recorded.

**Results:**

Most participants (77%) described inpatient sleep quality to be worse or much worse compared to their home environment. Noise (39%), checking of vital signs (33%) and light (24%) were most frequently identified as factors disrupting sleep. Binary logistic regression analysis demonstrated that men (OR 2.8, CI 1.1–7.4, *p* = 0.037) and those in shared rooms (OR 3.9, CI 1.4–10.9, *p* = 0.009) were more likely to be affected by noise. Younger patients (OR 0.92, CI 0.88–0.96, *p* < 0.001) and those in shared rooms (OR 8.5 CI 1.9–37.9, *p* < 0.001) were more likely to be affected by light.

**Conclusion:**

In conclusion, a high proportion of hospitalised respiratory patients on a medical ward reported poorer sleep quality compared to home due to operational interruptions and noise. Age, gender and room type further modified the sleep disruption. Future research should focus on whether strategies to reduce interruptions and noise will improve sleep quality and clinical outcomes.

## Introduction

Sleep is a restorative process that plays a role in healing and recovery from acute and chronic disease [1]. A significant proportion of patients experience poor sleep in hospital that may hinder recovery due to complications such as delirium, anxiety and mood disorders [2, 3]. Whilst many studies have focused on sleep in high-acuity environments, such as the intensive care unit, [4, 5] it is also important to characterise sleep disruption in the acute medical ward setting.

## Methods

**Fig. 1:**
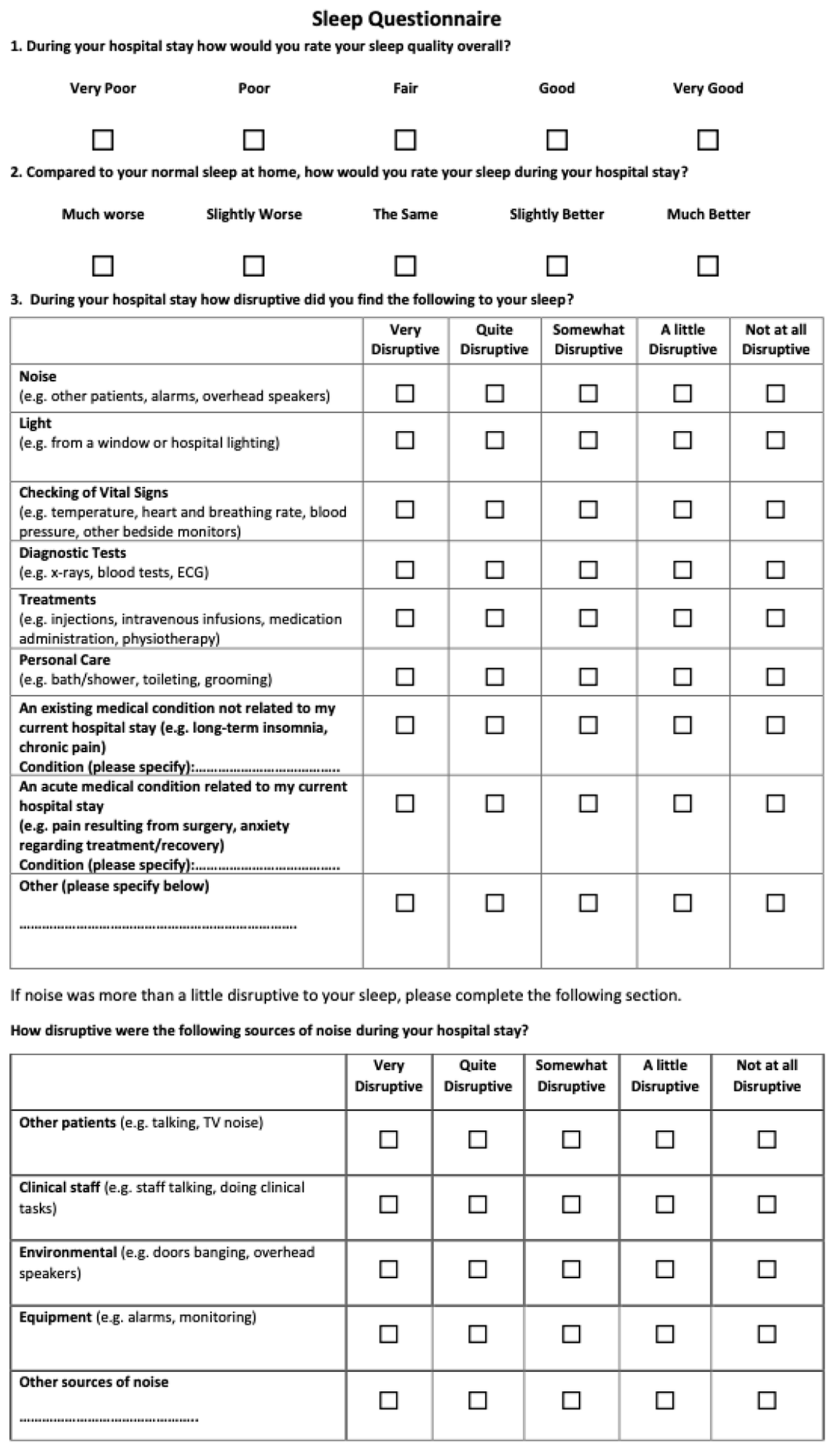
Survey questionnaire

Respiratory inpatients (*N* = 84) managed on a shared respiratory/ cardiology ward completed a 5-point Likert scale questionnaire (Fig. 1) at discharge, rating their sleep quality in hospital and factors which disrupted sleep, including sources of noise. Demographic data, room type (shared or individual) and clinical information, including hospital length of stay, were recorded. Normally distributed variables were compared using ANOVA and categorical variables using Chi-square tests. Binary logistic regression was used to assess the relationship between age, gender, room type and sleep disruption. The study was approved by the Eastern Health Ethics Committee.

## Results

Patient data, stratified by sleep quality rating, are presented in Table 1. Forty (47.6%) patients were male and the median age was 66 (IQR 52–79) years. 25% were admitted with an exacerbation of COPD and 44% were assigned to a shared room. Thirty-six (43%) reported good or very good sleep, whilst 14 patients (16%) rated sleep quality as poor or very poor. The remaining patients (41%) reported their sleep quality as fair. When comparing sleep quality in hospital to home, 65 (77%) patients reported worse or much worse sleep. Noise (39%), checking of vital signs (33%) and light (24%) were most frequently identified as factors disrupting sleep.

**Table 1:** Summary of characteristics and responses from respondents of survey questionnaire

Sleep quality rating	Very good/good	Fair	Poor /very poor	*p*-value
Male / female (% total)	16/20 (43)	17/17 (40)	8/6 (17)	N.S.
Age, yr (IQ range)	65 (51–80)	70 (52–81)	67 (49–72)	0.588
Single room, n (%)	24 (67)	16 (47)	7 (50)	N.S.
Length of stay, days (IQR range)	6.5 (3.0-10.7)	4.5 (3.0–8.0)	3.0 (2.0-5.7)	0.051
Sleep disorder, n (%)	13 (36)	6 (18)	3 (21)	N.S.
COPD exacerbation, n (%)	10 (28)	8 (24)	3 (21)	N.S.
Disruptors of Sleep:				
Noise (n[%])	8 (22)	17 (50)	8 (57)	0.019
Light (n[%])	5 [14]	9 [26]	6 [43]	N.S.
Vitals check (n[%])	6 [17]	14 [41]	8 [57]	0.011
Tests (n[%])	4 [11]	7 [21]	2 [14]	N.S.
Treatment (n[%])	5 [14]	10 [29]	2 [14]	N.S.
Personal Care (n[%])	1 [3]	2 [6]	0 [0]	N.S.
Existing medical condition (n[%])	12 [33]	4 [12]	4 [29]	N.S.
Acute medical condition(n[%])	13 [36]	14 [12]	9 [64]	N.S.

*N.S.: *not significant*

When comparing the groups stratified according to good, fair and poor sleep quality, there were no significant differences in age, gender, presence of underlying sleep disorder or room assignment. There was a significant difference in the proportion of patients across groups identifying checking of vital signs and noise as disruptive. The main sources of noise were the environment (38%), equipment (22%), and staff (19%). Length of stay was reduced in the poor sleep group (3.0 days [2.0-5.7] vs. 6.5 days [3.0-10.7], *p* = 0.051). Further review revealed 10 cases with fair to good sleep quality, where discharge was delayed due to pending results or procedures, psychosocial factors or prolonged management for pneumothorax. With exclusion of these cases, length of stay did not differ between the groups (*p* = 0.580).

Binary logistic regression analysis demonstrated that men (OR 2.8, CI 1.1–7.4, *p* = 0.037) and those in shared rooms (OR 3.9, CI 1.4–10.9, *P* = 0.009) were more likely to be affected by noise. Younger patients (OR 0.92, CI 0.88–0.96, *P* < 0.001) and those in shared rooms (OR 8.5 CI 1.9–37.9, *P* < 0.001) were more likely to be affected by light.

## Discussion and conclusion

Our study demonstrates that poor sleep quality is prevalent in respiratory patients admitted to an acute medical ward and that sleep is subjectively worse in hospital than at home. Noise and checking of vital signs are the most significant contributors to sleep disruption. We present a novel finding that age, gender and room type independently affect self-reported sleep quality. Men are more likely to be affected by noise, younger patients by light and those in shared rooms by both noise and light.

Our findings support previous studies demonstrating that sleep is reduced in hospital by environmental factors, including checking of vital signs, noise and light [6] and extends these findings to a cohort of respiratory inpatients on an acute medical ward. Previous non-randomised studies have shifted the measurement of vital signs till later in the morning to improve sleep [7]. Similar to our study, shared rooms have been reported to contribute to poorer sleep in hospital [8], noting modern hospital designs encourage the use of single rooms.

It is unclear why certain groups are more vulnerable to sleep disruption attributable to noise and light stimuli. We speculate that older patients may be less affected by light compared to their younger counterparts due to age-related reduction in light sensitivity and subsequent effects on melatonin suppression [9]. A 2014 study [10] assessed gender differences in noise perception and found that whilst women reported more noise annoyance, reduced sleep duration and quality was only seen in men, suggesting that women are more likely to adapt their behaviour to the noise exposure.

We note the limitations of our study, including the restricted cohort of respiratory inpatients on one ward. A broader sample across several wards and specialties may have produced more representative data, however it is challenging to control for confounding factors such as difference in ward environment, staff and procedures.

In conclusion, a high proportion of hospitalised respiratory patients on a medical ward reported poorer sleep quality compared to home due to operational interruptions and noise. Age, gender and room type further modified the sleep disruption. Future research should focus on whether strategies to reduce interruptions and noise will improve sleep quality and clinical outcomes. Findings from this study could be translated into clinical sleep modifications on the ward, such as eye masks for younger patients and ear plugs for males to improve sleep quality and, potentially, health outcomes.

## Data Availability

The data that support the findings of this study are not openly available due to reasons of sensitivity and are available from the corresponding author upon reasonable request. Data are located at Box Hill Hospital.

## Declarations

### Conference presentation

Australian Sleep Association “Sleep DownUnder”, Adelaide, Australia (November 2023).
